# Antimicrobial-Resistant *Escherichia coli* from Environmental Waters in Northern Colorado

**DOI:** 10.1155/2019/3862949

**Published:** 2019-02-18

**Authors:** Hannah B. Haberecht, Nora Jean Nealon, Jake R. Gilliland, Amethyst V. Holder, Connor Runyan, Renee C. Oppel, Hend M. Ibrahim, Link Mueller, Forrest Schrupp, Samuel Vilchez, Linto Antony, Joy Scaria, Elizabeth P. Ryan

**Affiliations:** ^1^Environmental and Radiological Health Sciences, Colorado State University, Fort Collins, CO 80523, USA; ^2^Graduate Program in Cell and Molecular Biology, Colorado State University, Fort Collins, CO 80523, USA; ^3^Department of Medical Biochemistry, Faculty of Medicine, University of Zagazig, Zagazig 44519, Egypt; ^4^Drake Water Reclamation Facility, Fort Collins, CO 80525, USA; ^5^Department of Microbiology and Parasitology, Universidad Nacional Autónoma de Nicaragua, León, Nicaragua; ^6^Department of Veterinary and Biomedical Sciences, South Dakota State University, Brookings, SD 57007, USA

## Abstract

Waterborne *Escherichia coli* are a major reservoir of antimicrobial resistance (AMR), including but not limited to extended-spectrum beta-lactamase (ESBL) and *Klebsiella pneumoniae* carbapenemase (KPC) mechanisms. This study quantified and described ESBL- and KPC-producing *E. coli* in Northern Colorado from sewer water, surface water, and influent and effluent wastewater treatment sources. Total detected bacteria and *E. coli* abundances, and the percentages that contain ESBL and/or KPC, were compared between water sources. Seventy *E. coli* isolates from the various waters had drug resistance validated with a panel of 17 antibiotics using a broth microdilution assay. The diverse drug resistance observed across *E. coli* isolates was further documented by polymerase chain reaction of common ESBL genes and functional relatedness by PhenePlate assay-generated dendrograms (*n*=70). The total *E. coli* abundance decreased through the water treatment process as expected, yet the percentages of *E. coli* harboring ESBL resistance were increased (1.70%) in surface water. Whole-genome sequencing analysis was completed for 185 AMR genes in wastewater *E. coli* isolates and confirmed the presence of diverse AMR gene classes (e.g., beta-lactams and efflux pumps) in isolate genomes. This study completed surveillance of AMR patterns in *E. coli* that reside in environmental water systems and suggests a role for integrating both phenotypic and genotypic profiling beyond ESBL and KPC mechanisms. AMR screening via multiple approaches may assist in the prevention of drug-resistant *E. coli* spread from waters to animals and humans.

## 1. Introduction

Antimicrobial-resistant (AMR) bacteria are ubiquitous in environmental waters, including oceans [1, 2], rivers [3, 4], lakes [5, 6], and sewer water [7, 8], and have even been recorded in drinking water sources [9–12]. Water systems harbor antibiotics, biocides, heavy metals, and other chemicals [13, 14] that naturally select for antimicrobial resistance within these waterborne microbial gene pools. *Escherichia coli* is abundant in water systems [15] and is a concerning reservoir for AMR in these locations [12, 16, 17]. A survey of AMR *E. coli* in the Netherlands showed 17.1% of ESBL *E. coli* isolated from river water and wastewater were reported as pathogenic, and of those pathogenic strains, approximately 84% exhibited resistance in up to three drug classes including beta-lactams, tetracyclines, and aminoglycosides [18]. In an estuary in Portugal, isolated *E. coli* was phylogenetically distributed into commensal and pathogenic groups, and bacteria in both groups were resistant to last-resort antibiotics, including carbapenems [19]. Given that antibiotics are conventionally used to treat *E. coli* infections in people and animals, improved AMR surveillance and a better understanding of how resistance spreads in environmental waters are warranted [20, 21].

Two major types of beta-lactam resistance include microbial production of extended-spectrum beta-lactamase (ESBL) and *Klebsiella pneumoniae* carbapenemase (KPC) enzymes [22]. These enzymes work via hydrolysis of the beta-lactam ring (ESBL) or via interference with antibiotic binding to penicillin-binding proteins (KPC) [23]. The Centers for Disease Control and Prevention classifies ESBL as a serious threat and KPC as an urgent threat to public health [24]. In the USA alone, ESBL resistance among Enterobacteriaceae, which includes *E. coli*, was reported to have caused approximately 1,700 deaths with medical costs per infection in excess of $40,000 US dollars [24]. KPC was attributed to 600 deaths in 2013 [24], and carbapenem drugs are considered a last-resort antibiotic for severe bacterial infections [25]. Furthermore, *E. coli* that express ESBL and/or KPC genes have been frequently shown to harbor resistance to other antibiotic classes [11, 26]. A thorough application of functional screening methods may be needed to comprehensively characterize the AMR phenotypes of waterborne *E. coli*.

There are multiple genes contributing to ESBL and KPC resistance, including *blaCTX-M*, *blaOXA*, *blaSHV* and *blaTEM*, and *blaKPC*, *blaVIM*, *blaIMP*, and *blaNDM* [27], all of which can be transferred to other species horizontally on plasmids [28]. Considering the potential of horizontal AMR gene transfer into reservoirs of human pathogens, increased attention is being given to the identification of AMR genes in bacteria isolated from environmental waters.

The objective of this study was to screen environmental water samples for AMR *E. coli*, to understand the phenotypic and genotypic resistance profiles of *E. coli* isolates, and to examine clonal relatedness of AMR *E. coli* strains across water systems. It was hypothesized that KPC and ESBL screening of environmental *E. coli* would be predictive of additional multidrug resistance mechanisms in these isolates, and that genotypic and phenotypic analysis would reveal patterns in drug resistance based on date and location of the water sources sampled. The cross-validation of the methodologies reported herein illustrates the depth by which multiple analysis platforms can be integrated to establish AMR profiles of *E. coli* across environmental waters.

## 2. Materials and Methods

### 2.1. Environmental Water Sampling Locations and Collection Procedures

Water samples were collected from Larimer County, Colorado, from May 2016 to April 2017. All water samples were collected with autoclaved Pyrex® wide mouth storage bottles (quantity of 1 liter). Following collection, all samples were immediately placed on ice and stored in a light-sensitive container until analysis, which occurred at least one hour post-collection. Remaining sample was then stored at 4°C. A map of sample collection locations is provided in supplementary information (Figure S1). Seventeen water samples were collected and classified as follows: sewer water, wastewater treatment plant (WWTP) influent, WWTP effluent, and surface water. Sewer water and WWTP influent were grouped as wastewater, and WWTP effluent and surface water were grouped as ambient water. Sewer water (*n*=6) was collected from five manholes downstream from a human hospital and also from area residents, businesses, and a university. WWTP-influent (*n*=3) and WWTP-effluent (*n*=2) samples were collected using the Hawk Composite Sampler (Hach, Loveland, CO) at the Drake Water Reclamation Facility, a conventional activated sludge treatment plant. WWTP-influent samples were collected before water treatment, and WWTP-effluent samples were collected after sulfur dioxide and chlorine treatment and prior to discharge into the Cache la Poudre River. Surface water (*n*=6) was collected from five sources along the Cache La Poudre River both upstream and downstream from the WWTP-effluent discharge, with one site being upstream of the city limits. Storage bottles were submerged midstream in surface water for river samples and from covered manholes for sewer water samples. WWTP-influent and WWTP-effluent samples were collected midflow from the Hawk Composite Sampler.

### 2.2. Microbiological Analysis of Total Detected Bacteria and Suspected *E. coli* from Environmental Water Samples

Seventeen independent sampling events between May 2016 and April 2017 were screened for total detected bacterial and *E. coli* abundance, as well as for ESBL and KPC resistance. Water samples were processed within 24 hours of acquisition. Samples were serially diluted in sterilized phosphate-buffered saline (1× PBS, pH 7.4, VWR, Radnor, PA) at room temperature (23°C) and subjected to membrane filtration through cellulose nitrate filters (0.45 *µ*m pore size; Millipore Sigma, Burlington, MA) for bacterial enumeration. With each sample, filtration was performed in triplicate at three different dilutions and membrane filters were placed on three different chromogenic agar types: CHROMagar Orientation™; CHROMagar ESBL™, which screens for ESBL-producing isolates; and CHROMagar KPC™ (DRG Diagnostics, Springfield, NJ), which screens for KPC-producing isolates. All agars, broths, and other liquid reagents were incubated for 24 hours and checked for sterility before use, and agar plates were stored along with inoculated plates to monitor for any contamination occurring during the filtering process. Additionally, contamination in sample dilutions was controlled for using sterilized and filtered phosphate-buffered saline (PBS) that was plated, incubated, and assessed for colony growth along with inoculated plates. Although CHROMagar KPC plates have higher sensitivity than KPC-screening agars [29, 30], bacterial growth on these plates is not an absolute conformation of KPC production [31, 32]. This is because resistance to carbapenems can be mediated by both KPC enzymes and efflux pumps [33], and consequently, a positive result will be referred to as “suspected KPC.” CHROMagar Orientation quantified total detected bacterial abundance and total *E. coli*. CHROMagar ESBL quantified ESBL-producing total detected bacteria and ESBL *E. coli*, and CHROMagar KPC quantified suspected KPC-producing total detected bacteria and *E. coli*. Plates were incubated at 37°C at 2–5% CO_2_ for 24–72 hours, until countable colonies were observed. Total colony-forming units (CFUs) per plate within the range of 15–300 were included for descriptive analysis. As per manufacturer instructions, species identifications were made using CFU color and morphology, where *E. coli* colonies were pink with a faint halo [34, 35]. *E. coli* counts were included in the abundance calculation if they were counted on a plate with at least 15 (lowest countable detection limit) and at most 300 colonies (highest countable detection limit). To compare total detected AMR bacteria and total AMR *E. coli* abundances between sampling locations, relative percentages of ESBL and suspected KPC resistance were calculated by dividing the number of ESBL or suspected KPC-positive *E. coli* or total detected bacteria by *E. coli* or total detected bacteria in each water source.

### 2.3. *E. coli* Purification, Isolation, and Species Confirmation

A spread plate-based screening of all water samples was used to collect *E. coli* isolates for more in-depth drug susceptibility and clonal relatedness analysis. *E. coli* isolates from each water sample were selected from 1 of the 3 CHROMagar plates (Orientation, ESBL, or KPC) based on the chromogenic appearance as pink colonies with a faint halo [34, 35]. Single colonies were isolated to MacConkey agar (Difco, BD Biosciences: Franklin Lakes, NJ) and incubated for 24–72 hours at 2–5% CO_2_, until visible colonies were observed. As per manufacturer instructions, isolates that appeared bright pink on MacConkey agar were defined as *E. coli* and were further isolated onto tryptic soy agar (Remel™, Lenexa, KS) plates and incubated overnight at 37°C. Single colonies were selected and inoculated into sterile tryptic soy broth (Remel™, Lenexa, KS) for 24–72 hours, until bacterial cell growth was observed. Blank tryptic soy broth was incubated alongside isolated cultures as a negative control, and any growth in noninoculated media blanks was addressed by repeating the assay by selecting new *E. coli* colonies from the original agar plate and inoculating them into new sterile media.

Potential *E. coli* isolates in tryptic soy broth were species-confirmed using matrix-assisted laser desorption ionization time of flight mass spectrometry (MALDI-TOF) at the Colorado State University Proteomics and Metabolomics Facility (CSU-PMF). Briefly, isolates were dissolved in 1 *µ*L of 70% formic acid on a Biotyper plate (Bruker Daltonics Inc© Billerica, MA), and the dried spots were mixed with 1 *µ*L of alpha-Cyano-4-hydroxycinnamic acid (HCCA) and analyzed in triplicate using the VITEK-MS™ (Biomerieux, USA). A 0.5 *µ*L Bacterial Test Standard (Bruker Daltonics Inc© Billerica, MA) was added as an internal control. Protein identities were assigned using a CSU-PMF internal library, where a 70% match of total mass-spectral peaks (score of >2.0) identified an isolate as *E. coli*. Seventy total isolates were confirmed as *E. coli*, including ESBL-producing (*n*=12), ESBL + suspected KPC-producing (*n*=35), and neither ESBL- nor KPC-producing (*n*=23) *E. coli*.

### 2.4. DNA Isolation and PCR Amplification of ESBL and KPC Genes from *E. coli* Isolates

To further establish *E. coli* ESBL resistance profiles, polymerase chain reaction (PCR) was performed on the 70 confirmed *E. coli* isolates for 3 genes (*blaOXA*, *blaCTX-M*, and *blaTEM*) representative of ESBL phenotypes. The following primer sequences were used during DNA amplification, where “F” indicates the sequence of the forward primer, “R” indicates the sequence of the reverse primer, and “bp” indicates the number of base pairs in the primer:

*blaOXA* (F: ACA CAA TAC ATA TCA ACT TCG C, R: AGT GTG TTT AGA ATG GTG ATC, 813 bp), *blaCTX-M* (F: ATG TGC AGY ACC AGT AAR GTK ATG GC, R: TGG GTR AAR TAR GTS ACC AGA AYC AGC GG, 593 bp), and *blaTEM.* (F: CGC CGC ATA CAC TAT TCT CAG AAT GA, R: ACG CTC ACC GGC TCC AGA TTT AT, 445 bp).

*E. coli* isolates were lysed in 100 *µ*L of Millipore water at 100°C for 1 hour using a Bio-Rad T100™ thermocycler (Bio-Rad Laboratories, Inc, California). Amplification was carried out by 2 *µ*L DNA, 10 pmol of each primer, and 12.5 *µ*l Emerald Amp® GT PCR Master Mix (Takara Bio Inc., Clontech, Japan) under previously described conditions [36]. The PCR conditions were as follows: 15 minutes of denaturation at 95°C (1 cycle), 30 seconds of denaturation at 94°C, 90 seconds of annealing at 62°C, and 1 minute of polymerization at 72°C (34 cycles), with a final extension at 72°C for 10 minutes. PCR products were analyzed on a 1.5% agarose gel (Bio-Rad, Hercules, CA) and visualized using 1 *µ*L of ethidium bromide (Thermo Fisher, Lafayette, CO). PCR-grade water or sterile autoclaved 1× PBS was used as a negative control. Bacterial isolates with confirmed presence of *blaOXA*, *blaCTX-M*, and *blaTEM* were included as positive controls.

### 2.5. Biochemical Analysis to Establish Clonal Relatedness across *E. coli* Isolates

*E. coli* profiled with PCR were screened for clonal relatedness using a PhP-RE plate (PhP-RE, PhPlate AB, Microplate Techniques, Stockholm, Sweden) following the manufacturer's instructions [37]. The system consisted of a 96-well plate coated with 11 carbon sources: cellobiose, lactose, rhamnose, deoxyribose, sucrose, sorbose, tagatose, D-arabitol, raffinose, gal-Lacton, and ornithine. Three hundred *µ*L of media containing a pH indicator (bromothymol blue 0.11% w/v) and proteose peptone (Sigma-Aldrich: St. Louis, MO) was combined with ∼1 mg of each bacterial isolate. After 1.5 hours, 12 *µ*L of inoculum was transferred into each substrate well. One row of substrate was not inoculated and served as a negative control. As an internal check for reproducibility, three replicate isolate pairs were grown on separate 96-well plates and were determined to have correlation values of 0.94 and above. Plates were incubated at 37°C, covered with light-sensitive material, and read at 620 nm on a BioTek Cytation™3 (BioTek Instruments: Winooski, VT) at 8 h, 24 h, and 48 h. Clonal relatedness was estimated using PhenePlate™ software (PhPlate AB), which examined variability in absorbance across each substrate and presented relatedness as a dendrogram. A cutoff value of <97.5% similarity in functional profiles defined isolates as clonally distinct from each other.

### 2.6. *E. coli* Isolates Tested for Antibiotic Susceptibility to 17 Antibiotics

Thirty-four *E. coli* isolates were further screened for antimicrobial resistance using a broth microdilution assay at the Colorado State University Veterinary Teaching Hospital-Diagnostic Medicine Center (VTH-DMC). The isolates were from sewer water (*n*=17), WWTP influent (*n*=10), WWTP effluent (*n*=2), and surface water (*n*=5), and were ESBL-producing (*n*=10), suspected ESBL + KPC-producing (*n*=1), or neither KPC- nor ESBL-producing (*n*=23) *E. coli*. Given the higher abundance of ESBL/KPC-positive *E. coli* detected in wastewater samples during membrane filtration, this analysis preferentially screened *E. coli* isolates from those water sources. The 17 antibiotics tested included amoxicillin/clavulanic acid, ampicillin, cefalexin, cefovecin, ceftiofur, chloramphenicol, enrofloxacin, gentamicin, imipenem, marbofloxacin, nitrofurantoin, piperacillin, tetracycline, tobramycin, trimethoprim/sulfamethoxazole, amikacin, and cefpodoxime. Minimum inhibitory concentrations (MIC) were calculated and categorized as susceptible, intermediate, or resistant based on standards set by the Clinical and Laboratory Standards Institute (CLSI) guidelines [38]. For each drug, percent resistance was calculated by dividing the number of resistant isolates by the total number of isolates screened with that drug.

### 2.7. Whole-Genome Sequencing of *E. coli* Isolates

Whole-genome sequencing was performed on a subset of *E. coli* isolated from WWTP-influent and sewer water samples. Twenty-five *E. coli* isolates were grown at 37°C at 2–5% CO_2_ for 24–48 hours in sterile tryptic soy broth (TSB), centrifuged at 4000× g for 10 minutes (Beckman Coulter Allegra X-14R, Indianapolis, IN, USA), and reconstituted in approximately 250 *µ*L of TSB. DNA was extracted using a DNeasy PowerSoil Kit (Qiagen, Valencia, CA) following manufacturer protocols and stored at −20°C until quality checking and quantification with a NanoDrop 2000 (Thermo Scientific, Lafayette, CO). Incubated sterile TSB media and sterile DNA extraction media were used as negative controls.

Whole-genome sequencing of DNA extracts was performed at the Animal Disease Research and Diagnostic Laboratory at South Dakota State University in Brookings, SD, using previously described methods [39]. Briefly, 0.3 ng/*µ*L of DNA from each isolate was processed using a Nextera XT DNA Sample Prep Kit (Illumina Inc., San Diego, CA), pooled together, and sequenced on an Illumina Miseq platform using a 2 × 250 paired end approach with V2 chemistry (Illumina Inc., San Diego, CA). Raw sequencing files were demultiplexed and converted to FASTQ files using Casava version 1.8.2 (Illumina Inc., San Diego, CA). The CLC Genomics workbench version 9.4 (Qiagen bioinformatics, Valencia, CA) trimmed and assembled sequences, and for each isolate, AMR genes were BLAST searched against 185 distinct gene sequences from ResFinder [40] and the Comprehensive Antibiotic Resistance Database [41]. Gene identities were made based on a minimum 85% sample gene identity match over 50% of the gene sequence length, as compared with the database entry of the gene.

## 3. Statistical Analysis

Total bacteria and total *E. coli* abundances were compared across the four water sample types using a Kruskal–Wallis nonparametric analysis of variance test with a Dunn's post-test to adjust for multiple comparisons. Ambient and wastewater were compared using a nonparametric Mann–Whitney test. A *p*–value of *p* < 0.05 was defined as a statistically significant difference between water sources, and these values are reported alongside source abundance fold differences in the results below.

## 4. Results and Discussion

### 4.1. Total Detected Bacterial and *E. coli* Abundances through Wastewater Treatment Process

Total detected bacterial and *E. coli* abundances across environmental waters tested in 17 independent sampling events are depicted in Table 1. The negative control plates included for each filtered sample did not contain microbial growth. Filter plates from each source that had CFU counts between 15 and 300 and were included in source abundance. Of 460 total filter plates counted, a total of 216 plates were excluded from analysis, including 34 that exceeded a count of 300. To estimate relative bacterial density, total detected bacterial abundance and *E. coli* abundance were compared across sewer water, WWTP-influent, WWTP-effluent, and surface water locations. Total detected bacteria ranged from 2.1 + 04 CFU/100 mL in surface water to 3.4*E* + 08 CFU/100 mL in WWTP influent. Among wastewater, WWTP influent had 1.7-fold greater total detected bacterial abundance than sewer water (3.4*E* + 08 CFU/100 mL and 2.1*E* + 08 CFU/100 mL, respectively), though this difference was not significantly significant. Elevated levels of bacteria in WWTP-influent samples could be due to the convergence of different sewer water sources into a common WWTP (Figure S1). Wastewater (sewer and WWTP influent) had 3221-fold higher (*p* < 0.001) total detected bacterial abundance than ambient water (WWTP effluent and surface water). Decreases in total detected bacterial abundance after wastewater treatment have been previously reported in other microbial wastewater treatment sampling investigations [42–44], suggesting that this studied wastewater treatment plant functions in a similar manner to previously examined plants.

As a percentage of total detected bacteria, *E. coli* was the highest in sewer water at 37%, detected at lower levels in WTTP influent (22%) and surface water (14%), and was lowest in WWTP effluent (2.4%) (Table 1). *E. coli* abundance followed similar trends to total detected bacterial abundance, with the greatest total *E. coli* found in wastewater (averaging 1.5*E* + 08 CFU/100 mL) than in ambient water (averaging 3.3*E* + 03 CFU/100 mL). The observed elevations in *E. coli* in sewer water and WWTP influent could be due to the proximity of fecal contamination from humans and animals, as reported previously [20, 21, 45–48].

### 4.2. Total and *E. coli* ESBL and KPC Abundance Varied across Environmental Water Sources

**Figure 1:**
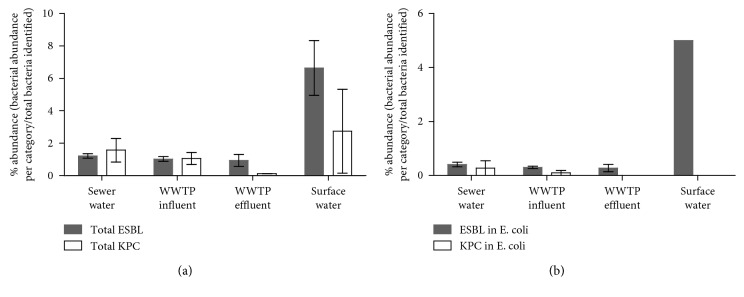
Relative percentages of ESBL and suspected KPC in total detected bacteria and *E. coli* sampled from environmental waters. Relative percentages of antimicrobial-resistant bacteria from (a) total detected bacterial colonies and (b) *E. coli* collected from membrane filtration across 17 respective environmental water sources. Error bars, representing standard deviation, were generated from each numerator value (individual ESBL/KPC bacterial abundance counts per source) divided by the average of total detected plated bacteria per source. ESBL = extended-spectrum beta-lactamase (grey bars), KPC = *Klebsiella* pneumoniae carbapenemase (white bars), and WWTP = wastewater treatment plant.

Each water sample was screened for total ESBL and KPC resistance to estimate levels in sampled bacteria. The abundances (absolute counts) and relative percentages (resistant *E. coli* or total detected bacteria divided by total *E. coli* or total detected bacteria) of ESBL and suspected KPC bacteria are depicted by water source in Table 1 and Figure 1. ESBL and suspected KPC resistance were present in all water sources tested for total detected bacteria. Sewer water (2.5*E* + 06 CFU/100 mL) and WWTP influent (3.5*E* + 06 CFU/100 mL) together had a 2146-fold higher (*p* < 0.0001) ESBL-positive bacterial abundance than the ambient sources, which were 1.4*E* + 03 CFU/100 mL (WWTP-effluent) and 1.4*E* + 03 CFU/100 mL (surface water). Suspected KPC bacterial abundance in wastewater was 8847-fold higher (*p* < 0.0001) (3.4*E* + 06 CFU/100 mL WWTP-influent, 3.2*E* + 06 CFU/100 mL sewer water) than that in ambient water (5.8*E* + 02 CFU/100 mL surface water, 1.7*E* + 02 CFU/100 mL WWTP-effluent). The relative percentage of ESBL in total detected bacteria ranged from 0.93% (WWTP-effluent) to 6.6% (surface water) and the relative percentage of suspected KPC ranged from 0.11% (WWTP-effluent) to 2.7% (surface water) (Figure 1(a)).

Abundance of ESBL *E. coli* in wastewater (3.1*E* + 05 CFU/100 mL sewer water, 2.3*E* + 05 CFU/100 mL WWTP-influent) was 23318-fold higher (*p* < 0.01) than that in ambient water (1.0*E* + 01 CFU/100 mL WWTP-effluent, 5.0*E* + 01 CFU/100 mL surface water). Similarly, abundance of suspected KPC *E. coli* in wastewater (2.1*E* + 05 CFU/100 mL sewer water, 7.8*E* + 04 CFU/100 mL WWTP-influent) was higher, though not statistically significant than suspected KPC *E. coli* abundance in ambient water (*E. coli* in WWTP effluent and surface water were below the limit of detection). Relative percentages of ESBL-positive *E. coli* ranged from 0.28% in WWTP effluent to 1.7% in surface water, and suspected KPC-positive *E. coli* was below detectable limits in WWTP effluent and surface water and greatest in sewer water (0.28%) (Figure 1(b)). *E. coli* represented 8.9% of total ESBL and 4.2% of total suspected KPC abundance of all bacteria screened.

Abundance of drug-resistant total detected bacteria and *E. coli* decreased between wastewater and ambient water samples and specifically between WWTP-influent and WWTP-effluent samples. The relative percentage of KPC also decreased between WWTP-influent and WWTP-effluent sources in both *E. coli* and total detected bacteria. It is noteworthy that other wastewater treatment studies have reported increased relative percentage of KPC-producing bacteria following wastewater treatment [49–51]. The sludge found in many WWTPs is thought to be an antibiotic resistance gene reservoir [52–54], and pressures put onto bacteria during the treatment process can include contact with residual antibiotics [55], driving the transfer and development of resistance. Consequently, the factors contributing to the reduced abundances of suspected KPC *E. coli* between WWTP influent and WWTP effluent in this plant warrant investigation for application to other water treatment systems.

Surface water had the greatest relative percentage of ESBL *E. coli* and the greatest relative percentage of ESBL and suspected KPC-positive total detected bacteria. The proximity of surface water sampling sites to human recreational and agricultural inputs may explain this trend. Supporting this explanation, multiple studies have reported a transfer of ESBL and KPC-expressing bacteria from people and animals into water systems [11, 56, 57].

### 4.3. Phenotype and Genotype Profiles Moderately Separate *E. coli* Isolates by Source

**Figure 2:**
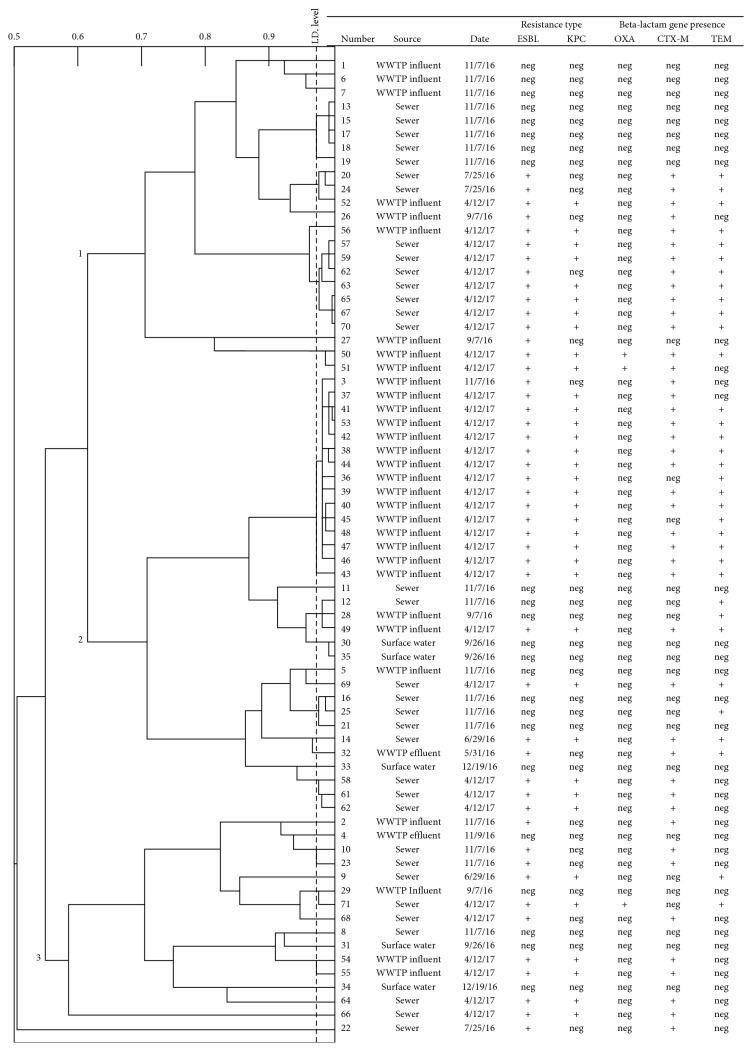
Clonal relatedness of *E. coli* isolates (*n*=70) from environmental sources using a functional biochemical analysis and PCR verification of beta-lactam resistance gene presence. Identity “ID” levels are to the left of the dotted line, which represents a sensitivity of 0.975. Branches to the right of this line represent isolates that are too similar to distinguish. Bold numbers on branches (1, 2, 3) refer to three major clusters of isolates identifiable from the Pheneplate assay. Isolate numbers represent the unique identifiers assigned to each *E. coli* isolate throughout this study. WWTP = water treatment plant. Date = isolation date. ESBL = extended-spectrum beta-lactamase and KPC: *Klebsiella pneumoniae* carbapenamase. *OXA*, *CTM-M*, and *TEM* were the three beta-lactamase genes assessed with PCR, where “+”: gene present and “neg”: gene absent.

Previous studies have inspected ESBL and KPC gene reservoirs to understand their spread through environmental waters [12, 58, 59], yet additional research is needed to elucidate patterns in *E*. *coli* resistance based on sampling location and date. 70 *E. coli* isolates with confirmed identity by MALDI-TOF were examined using a PhenePlate assay, which uses patterns in carbon source metabolism to describe clonal relatedness across bacterial isolates. The dendrogram of clonal relatedness for these selected *E. coli* isolates is shown in Figure 2.

PhenePlate analysis generated a dendrogram showing 3 major branches of clonal relatedness and was able to separate 38 functionally distinct isolates. Branch 1 contained isolates exclusively from wastewater sources and branches 2 and 3 included bacteria from both waste water and ambient water locations. PhenePlate analysis was previously applied to separate *E. coli* by site, whereby this approach exhibited varying degrees of sensitivity [60, 61]. Each branch in Figure 2 contained isolates collected from multiple sampling dates, though sample dates do show a small degree of clustering. These results suggest functional differences in carbon source metabolism alone are not sensitive enough to clonally distinguish environmental *E. coli* by collection site and date.

In an attempt to further distinguish the *E. coli*, each isolate examined with PhP-RE was profiled for the presence of three commonly expressed genes conferring ESBL and KPC resistance, *blaOXA*, *blaCTX-M*, and *blaTEM* through PCR (expression profiles across isolates are shown in Figure 2). All PCR-negative controls were determined to be free of contamination, and all positive controls displayed the expected band size patterns (data not shown). High prevalences of *blaOXA*, *blaCTX-M*, and *blaTEM* have been reported in clinically resistant strains of *E. coli* in Colorado [62]. Dendrogram branching patterns show *blaCTX-M* and *blaTEM* across all sample types and collection dates. Other studies in Turkey and Austria, which examined AMR in waterborne *E. coli* before and after wastewater treatment, similarly observed that *blaCTX-M* was the most abundant ESBL-associated gene among environmental *E. coli* [63, 64]. The presence of *blaCTX-M* was independent of water source and was not indicative of clonal relatedness among isolates [65, 66]. The apparent prevalence of *blaCTX-M* across 3 branch points and water sources may also be explained by the different variants of this gene, including *blaCTX-M-1*, *blaCTX-M-2*, *blaCTX-M-8*, *blaCTX-M-9*, and *blaCTX-M-25*, which are present in environmental, human, and animal sources [67], but were not distinguished during PCR analysis herein. *blaOXA* was only detected in wastewater isolates collected during April 2017, and thus merits further attention as a marker that could distinguish antimicrobial *E. coli* between wastewater and ambient water sources. This is supported by previous investigations, where *blaOXA* presence was found to differentiate beta-lactam-resistant *E. coli* across different water sources [36, 68]. A greater understanding of *E. coli* ESBL resistance distinctions could be valuable in WWTP design and could help to direct wastewater treatment to continue elimination of *blaOXA* resistance from bacterial populations. Furthermore, despite some separation of ambient and wastewater samples, the integrated PhenePlate and PCR analysis was not sensitive enough to distinguish wastewater *E. coli* from each other, suggesting that genotypic and phenotypic variables other than carbon source metabolism and presence of common beta-lactam genes are needed to better characterize clonal relationships.

### 4.4. Broth Microdilution-Expanded *E. coli*-Resistant Phenotypes from Environmental Waters

To cross-validate the ESBL and suspected KPC-resistant phenotypes detected during spread plating of *E. coli*, and to understand if screening for additional classes of AMR could better distinguish isolates, a broth microdilution assay was performed on a subset of the MALDI-TOF-confirmed *E. coli* isolates. Given the higher abundance of ESBL and suspected KPC *E. coli* detected in wastewater compared with ambient water, sewer water (*n*=17) and WWTP-influent (*n*=10) samples were preferentially screened in this assay. Prior to broth microdilution testing, all of the negative control samples incubated along with the inoculated *E. coli* isolates were determined to be free of bacterial growth. Results from broth microdilution assay are shown in Table 2, which displays percent drug resistance of *E. coli* by sample source.  Table S1 provides a breakdown of resistance profiles for each isolate examined.

Diversity of drug resistance was seen between and within water sources (Table S1) A diverse array of resistance patterns across isolates may reflect multiple, independent horizontal gene transfers along isolate lineages [69]. This reasoning is supported in past studies that reported an escalation in the rate of horizontal gene transfer among *E. coli* over the last few decades, including genes responsible for ESBL and other resistance mechanisms [70, 71]. Broth microdilution is the CLSI-approved and conventional method used to screen for AMR in medical settings [72]. Broth microdilution results supported that screening *E. coli* for resistance to multiple drug classes can improve the capacity to distinguish closely related isolates from each other. The successful application of this assay to environmental samples collected herein emphasizes its utility as part of a harmonized surveillance approach for identifying shared relatedness of AMR in people, animals, and the environment.

### 4.5. Whole-Genome Sequencing for Isolate Resistance Profiles and the Spatial and Temporal Relationship of Resistance Genes

**Figure 3:**
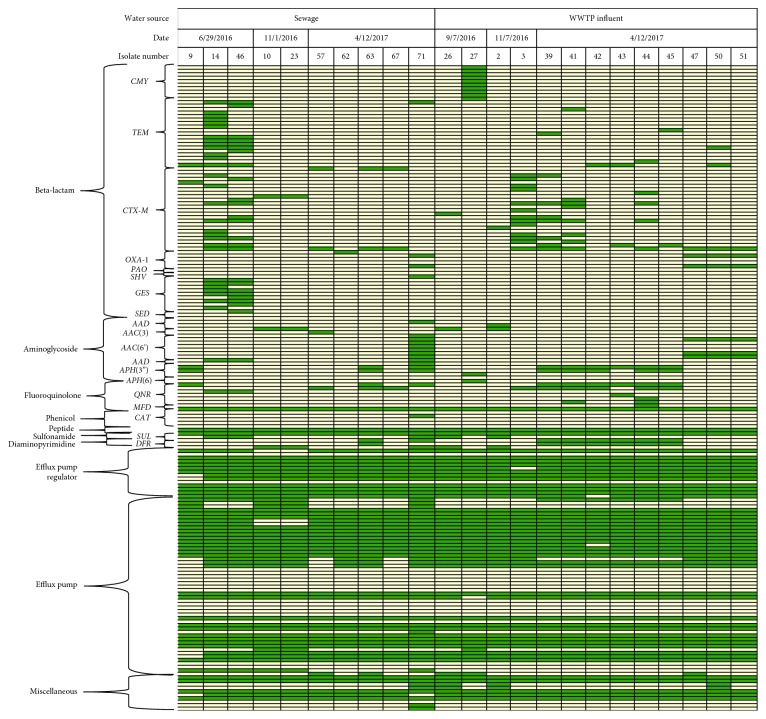
Whole-genome sequencing distinguished sewer water and WWTP-influent *E. coli* across sample type and collection date. AMR profiles of wastewater E. coli isolates (*n* = 21) are organized by sample type (sewer water versus wastewater treatment plant influent) and are further categorized by sample collection date. Antimicrobial function and subclass of gene family names were used to group resistance genes. For each isolate, green boxes indicate gene presence and beige boxes indicate gene absence. Isolate #: the unique identifier assigned to each E. coli isolate with MALDI-TOF confirmation.

While the integrated PhenePlate and PCR analysis supported differences in gene presence between wastewater and ambient samples, it was not sensitive to distinguish individual wastewater isolates from each other. Given the large variability in the antibiotic resistance profiles of wastewater *E. coli* observed during broth microdilution, whole-genome sequencing was performed on a subset (*n*=21) of wastewater isolates. The objective of whole-genome sequencing was to evaluate if deeper sequencing could reveal spatial and temporal differences in resistance patterns among these closely related *E. coli*. All blank media and DNA extraction reagents were confirmed to be free of contamination (data not shown), supporting that the downstream analysis was sensitive to differences across *E. coli* isolates. The results depicted in Figure 3 categorizes *E. coli* by sample type, date, and gene classes. A comprehensive table including each gene profiled is included in Table S2.

Of the 185 genes screened, 154 were identified across all isolates and included 66 that conferred resistance to beta-lactams, 18 to aminoglycosides, 7 to fluoroquinolones, 1 to chloramphenicol, 7 to peptides, 2 to sulfonamides, 2 to diaminopyrimidines, 12 to efflux pump regulators, 36 to efflux pumps, and 9 miscellaneous resistance genes involved in the inactivation and alternation of bacterial enzymes. This genetic profiling cross-validated and expanded the information available for *E. coli* isolates with AMR profiles established through ESBL/KPC spread plating and PCR.

Whole-genome sequencing revealed differences across wastewater *E. coli* isolates, most notably in beta-lactam genes that were dependent on water sample source and collection date. Across water samples, 51 beta-lactam resistance genes (broken down as 29 sewer water and 22 WWTP influent) distinguished sewer water and WWTP-influent isolates. These included multiple variants of *tem* and *ges* genes unique to sewer water isolates and *cmy* and *ctx-m* genes unique to WWTP-influent isolates. When examining sewer water *E. coli* across sampling dates, 43 beta-lactam genes were unique to one sampling date, including 37 among 6/29/16 isolates, 1 among isolates dated 11/7/16, and 5 among isolates dated 4/12/17, and included multiple *tem*, *ges*, and *ctx-m* gene variants. For WWTP-influent *E. coli*, 25 beta-lactam resistance genes were specific to sample collection dates. There were 10 *cmy* variants in samples dated 9/7/16, 6 *ctx-m* variants in samples dated 11/7/16, and 9 genes, primarily *tem* variants, that were unique to *E. coli* dated 4/12/17. All of these gene variants have been associated with drug-resistant *E. coli* infections in people [73–75], emphasizing that they should be closely monitored for spread into the environment. These results suggest the potential connection between AMR gene presence and time of year and support that temporal screening of AMR genes should be considered in future surveillance programs.

Furthermore, whole-genome sequencing provided additional insight into whether *E. coli* that grew on KPC plates were KPC-producing isolates. Although no carbapenemase-encoding genes, including *ndm-1*, *oxa-48*, and *vim* family genes [76–78], were detected in isolates, *E. coli* harbored multiple efflux pump genes. Efflux pump genes (35 sewer water and 31 WWTP influent) and 12 efflux pump regulator genes (12 sewer water and 12 WWTP-influent) were detected across *E. coli*. Efflux pumps represent one mechanism of resistance to carbapenem drugs [27] and provide supporting rational for why isolates may have tested positive for KPC despite the lack of carbapenemase-encoding genes. It is possible that these *E. coli* isolates contain KPC gene variants that are not currently recognized by standard AMR gene databases, and therefore highlights the need for expanded KPC resistance gene indexing.

This study provides a framework for analysis by which new investigations can enhance the statistical power of studies to characterize AMR in other geographic locations, and with potential utility across other matrices for testing, such as human and animal gastrointestinal tracts, agricultural systems, and food production systems. Screening samples for AMR via standardized detection and monitoring will improve needed comparisons between surveillance programs [79].

## 5. Conclusions

This study illustrates the landscape of *E. coli* AMR across various water sources in Northern Colorado and utilizes an array of methodologies for AMR validation. Total detected bacteria and *E. coli* abundances were shown to decrease through the water reclamation process, validating the effective clearance of microbes from human water sources by the wastewater treatment plant. The decreased *E. coli* abundance in ambient water was coupled with an increased relative percentage of ESBL/suspected KPC resistance in total detected bacteria and ESBL *E. coli *in surface water. The prevalence of ESBL/KPC-resistant *E. coli* in surface water warrants deeper evaluation of potential antimicrobial inputs to rivers.

Broth microdilution further characterized drug resistance in isolates across water sources and validated *E. coli* ESBL/KPC phenotypes. Identification of beta-lactam resistance in *E. coli* that initially tested negative for ESBL/suspected KPC resistance supports that a multi-platform screening approach may be necessary for accurate characterization of AMR landscapes in environmental water sources. The increased prevalence of multidrug-resistant isolates in WWTP influent as compared with sewer water illustrates how converging water sources can increase levels of drug-resistant in organisms entering wastewater treatment plants.

An integrated PhenePlate/PCR analysis was applied to understand the relationships between water types and diverse *E. coli* AMR profiles with respect to sampling location and date. PhenePlate analysis alone could not distinguish wastewater and ambient water *E. coli* and suggests that this single measurement of carbon source metabolism is not sensitive to distinguish some environmental water *E. coli* from each other. However, the coupling of PCR for common beta-lactam resistance genes, such as *blaOXA*, does support its use as a potential integrated test to distinguish waste and ambient water *E. coli.*

Whole-genome sequencing applied to wastewater *E. coli* validated the resistance phenotypes and genotypes observed in the ESBL/suspected KPC screening, broth microdilution, and PCR analyses, and was effective at separating isolates both by sample type (sewage versus WWTP-influent) and by collection date. Sequencing results supported that resistance to beta-lactam and aminoglycoside antimicrobials are major contributors to these geographic and temporal differences. Additionally, the clustering of isolates by source and collection date supports the integrity of bacterial samples and the unlikelihood of sample contamination. These results warrant investigation into the development of applications and more sensitive screenings of beta-lactams and aminoglycosides that can both predict and understand how antimicrobial resistance landscapes can change within and across environments over time, as well as from waste and ambient water sources.

The sample sizes used to conduct research on these field isolates were small with respect to seasonality, yet the cross-validation of the methodologies reported herein illustrates the depth by which multiple analysis platforms could be integrated to establish AMR profiles of *E. coli* across environmental water types. This study provides a framework by which investigators can standardize analyses, in larger, statistically powered studies, and specifically to characterize AMR landscapes across geographically diverse locations. The integrated use of these assays also has relevance to other matrices including human and animal gastrointestinal tracts, agriculture, and food production systems.

## Figures and Tables

**Table 1:** Relative abundances of total detected bacteria and *E. coli* by water source and by ESBL and KPC production.

Source	Sampling events	Total detected bacteria (CFU/100 mL)			*Escherichia coli* (CFU/100 mL)		
		All	ESBL	KPC	All	ESBL	KPC
Sewer water	6	2.1*E* + 08^b,c^ (2.8*E* + 08)	2.5*E* + 06^b,c^ (1.3*E* + 06)	3.2*E* + 06^b,c^ (1.5*E* + 06)	7.6*E* + 07^b,c^ (1.2*E* + 08)	3.1*E* + 05^b^ (2.6*E* + 05)	2.1*E* + 05 (2.1*E* + 05)

WWTP influent	3	3.5*E* + 08^d,e^ (3.2*E* + 08)	3.5*E* + 06^d,e^ (1.9*E* + 06)	3.6*E* + 06^d,e^ (1.3*E* + 06)	7.7*E* + 07^d,e^ (1.5*E* + 08)	2.3*E* + 05^d^ (1.1*E* + 05)	7.8*E* + 04 (7.0*E* + 04)

WWTP effluent	2	1.5*E* + 05^b,d^ (2.2*E* + 05)	1.4*E* + 03^b,d^ (1.4*E*+ 03)	1.6*E* + 02^b,d^ (1.3*E* + 02)	3.6*E* + 03^b,d^ (4.3*E* + 03)	100^d,b^ (102)	BDL

Surface water	6	2.1*E* + 04^c,e^ (3.8*E* + 04)	1.4*E* + 03^c,e^ (1.9*E* + 03)	5.8*E* + 02^c,e^ (5.5*E* + 02)	2.9*E* + 03^c,e^ (4.7*E* + 03)	500 (0)	BDL

Data represent colony counts collected during membrane filtration; all data are presented as mean (±standard deviation). Abundance was measured as colony-forming units/100 mL. Plates with counts between 15 and 300 total CFUs were considered for analysis. CFU = colony-forming unit. BDL = below detection limit. ESBL = extended-spectrum beta-lactamase. KPC = *Klebsiella pneumoniae* carbapenemase. WWTP = wastewater treatment plant. Statistical differences (*p* < 0.05) are noted between water sources with the following letters: ^a^sewer water and WWTP influent, ^b^sewer water and WWTP effluent, ^c^sewer water and surface water, ^d^WWTP influent and WWTP effluent, and ^e^WWTP influent and surface water.

**Table 2:** Percent resistance by *E. coli* to a panel of different antibiotics and across different water sources.

	*E. coli* source						
Drug class	Antibiotics	Sewer water (*n*=17) (%)	WWTP influent (*n*=10) (%)	Wastewater (sewer + influent) (*n*=27) (%)	WWTP effluent (*n*=2) (%)	Surface water (*n*=5) (%)	Ambient water (effluent + surface) (*n*=7) (%)
Aminoglycoside	Amikacin	0	0	0	0	0	0
	Gentamicin	11.7	0	7.40	50.0	0	14.2
	Tobramycin	5.88	0	3.70	50.0	0	14.2

Beta-lactam	Amoxicillin/clavulanic acid	29.4	40.0	33.3	50.0	0	14.2
	Ampicillin	41.1	50.0	44.4	50.0	0	14.2
	Cefalexin	35.2	40.0	37.0	50.0	0	14.2
	Cefovecin	35.2	40.0	37.0	50.0	0	14.2
	Cefpodoxine	35.2	40.0	37.0	50.0	0	14.2
	Ceftiofur	23.5	40.0	29.6	50.0	0	14.2
	Imipenem	29.4	40.0	33.3	50.0	0	14.2
	Piperacillin	41.1	50.0	44.4	50.0	0	14.2

Chloramphenicol	Chloramphenicol	5.88	10.0	7.40	0	0	0

Fluoroquinolone	Marbofloxacin	11.7	0	7.40	0	0	0
	Enrofloxacin	11.7	10.0	11.1	0	0	0

Nitrofuran	Nitrofurantoin	0	0	0	0	0	0
Tetracycline	Tetracycline	23.5	10.0	18.5	50.0	0	14.2
Sulfonamide	Trimethoprim/sulfamethoxazide	0	20.0	7.40	0	0	0

Percentage of *E. coli* isolates resistant to 17 drugs (5 drug classes) was calculated by dividing total *E. coli* resistant for a given drug by total *E. coli* examined in a source type. WWTP: wastewater treatment plant.

## Data Availability

The whole-genome sequences of all 23 E. *coli* isolates were submitted to the NCBI Sequence Read Archive (NCBI SRA). However, only 22 of them have been assigned with SRA numbers. Isolate NCBI SRA identification numbers are presented in Supplementary Dataset 1.

## Supplementary Materials

Supplementary Materials Figure S1: a map of Fort Collins, Colorado, and the surrounding area with sampling locations of environmental waters. Table S1: AMR profiles of the individual *E. coli* isolates examined with broth microdilution. Table S2: an expanded whole-genome sequence table that details all genes detected across wastewater *E. coli* isolates.

## References

[1] Hatosy S. M., Martiny A. C. (2015). the ocean as a global reservoir of antibiotic resistance genes. *Applied and Environmental Microbiology*.

[2] Moore D. F., Guzman J. A., McGee C. (2008). Species distribution and antimicrobial resistance of enterococci isolated from surface and ocean water. *Journal of Applied Microbiology*.

[3] Marti E., Jofre J., Balcazar J. L. (2013). Prevalence of antibiotic resistance genes and bacterial community composition in a river influenced by a wastewater treatment plant. *PLoS One*.

[4] Ronald J. A., Brena M., Melissa M. (2002). Antibiotic resistance of gram-negative bacteria in rivers, United States. *Emerging Infectious Disease Journal*.

[5] Pang Y.-C., Xi J.-Y., Li G.-Q., Shi X.-J., Hu H.-Y. (2015). Prevalence of antibiotic-resistant bacteria in a lake for the storage of reclaimed water before and after usage as cooling water. *Environmental Science: Processes & Impacts*.

[6] Rosas I., Salinas E., Martínez L. (2015). Characterization of *Escherichia coli* isolates from an urban lake receiving water from a wastewater treatment plant in Mexico city: fecal pollution and antibiotic resistance. *Current Microbiology*.

[7] Blaak H., van Hoek A. H. A. M., Veenman C. (2014). Extended spectrum β-lactamase- and constitutively AmpC-producing *Enterobacteriaceae* on fresh produce and in the agricultural environment. *International Journal of Food Microbiology*.

[8] Rodriguez-Mozaz S., Chamorro S., Marti E. (2015). Occurrence of antibiotics and antibiotic resistance genes in hospital and urban wastewaters and their impact on the receiving river. *Water Research*.

[9] Martin M. S., Santos I. C., Carlton D. D., Stigler-Granados P., Hildenbrand Z. L., Schug K. A. (2018). Characterization of bacterial diversity in contaminated groundwater using matrix-assisted laser desorption/ionization time-of-flight mass spectrometry. *Science of the Total Environment*.

[10] Sharma B., Parul fnm, Verma A. K. (2017). Occurrence of multidrug resistant *Escherichia coli* in groundwater of Brij region (Uttar Pradesh) and its public health implications. *Veterinary World*.

[11] Diab M., Hamze M., Bonnet R., Saras E., Madec J.-Y., Haenni M. (2018). Extended-spectrum beta-lactamase (ESBL)- and carbapenemase-producing *Enterobacteriaceae* in water sources in Lebanon. *Veterinary Microbiology*.

[12] Li S., Zhu Z. C., Wang L., Zhou Y. F., Tang Y. J., Miao Z. M. (2015). Prevalence and characterization of extended-spectrum beta-lactamase-producing *Enterobacteriaceae* in spring waters. *Letters in Applied Microbiology*.

[13] Baquero F., Martínez J.-L., Cantón R. (2008). Antibiotics and antibiotic resistance in water environments. *Current Opinion in Biotechnology*.

[14] Berendonk T. U., Manaia C. M., Merlin C. (2015). Tackling antibiotic resistance: the environmental framework. *Nature Reviews Microbiology*.

[15] Jang J., Hur H.-G., Sadowsky M. J., Byappanahalli M. N., Yan T., Ishii S. (2017). Environmental *Escherichia coli*: ecology and public health implications-a review. *Journal of Applied Microbiology*.

[16] Egervärn M., Englund S., Ljunge M. (2017). Unexpected common occurrence of transferable extended spectrum cephalosporinase-producing *Escherichia coli* in Swedish surface waters used for drinking water supply. *Science of the Total Environment*.

[17] Osinska A. (2017). the prevalence and characterization of antibiotic-resistant and virulent *Escherichia coli* strains in the municipal wastewater system and their environmental fate. *Science of the Total Environment*.

[18] Franz E. (2015). Pathogenic *Escherichia coli* producing Extended-Spectrum beta-Lactamases isolated from surface water and wastewater. *Scientific Reports*.

[19] Pereira A., Santos A., Tacão M., Alves A., Henriques I., Correia A. (2013). Genetic diversity and antimicrobial resistance of *Escherichia coli* from Tagus estuary (Portugal). *Science of the Total Environment*.

[20] (2011). *Guidelines for Drinking-Water Quality 2011*.

[21] (2012). *Recreational Water Quality Criteria*.

[22] Bush K. (2010). Alarming *β*-lactamase-mediated resistance in multidrug-resistant *Enterobacteriaceae*. *Current Opinion in Microbiology*.

[23] Page M. G. (2012). Beta-lactam antibiotics. *Antibiotic Discovery and Development*.

[24] CDC (2013). *Antibiotic Resistance Threats in the United States, 2013*.

[25] Hawkey P. M., Livermore D. M. (2012). Carbapenem antibiotics for serious infections. *BMJ*.

[26] Baudry P. J., Nichol K., DeCorby M. (2008). Comparison of antimicrobial resistance profiles among extended-spectrum-beta-lactamase-producing and acquired AmpC beta-lactamase-producing *Escherichia coli* isolates from Canadian intensive care units. *Antimicrobial Agents and Chemotherapy*.

[27] van Duin D., Doi Y. (2017). the global epidemiology of carbapenemase-producing *Enterobacteriaceae*. *Virulence*.

[28] Paterson D. L., Bonomo R. A. (2005). Extended-spectrum *β*-lactamases: a clinical update. *Clinical Microbiology Reviews*.

[29] Esther J., Edwin D. (2017). Prevalence of carbapenem resistant non-fermenting gram negative bacterial infection and identification of carbapenemase producing NFGNB isolates by simple phenotypic tests. *Journal of Clinical and Diagnostic Research*.

[30] Garcia-Quintanilla M., Poirel L., Nordmann P. (2018). Chromagar mSuperCARBA and RAPIDEC^®^ Carba NP test for detection of carbapenemase-producing *Enterobacteriaceae*. *Diagnostic Microbiology and Infectious Disease*.

[31] Bialvaei A. Z., Kafil H. S., Asgharzadeh M. (2016). Current methods for the identification of carbapenemases. *Journal of Chemotherapy*.

[32] Hinić V., Amrein I., Stammler S. (2017). Comparison of two rapid biochemical tests and four chromogenic selective media for detection of carbapenemase-producing gram-negative bacteria. *Journal of Microbiological Methods*.

[33] Arnold R. S., Thom K. A., Sharma S. (2011). Emergence of *Klebsiella pneumoniae* carbapenemase (KPC)-Producing bacteria. *Southern Medical Journal*.

[34] Al Anbgi N. (2016). Isolate and identification some bacterial causes of lung abscessesheep by chromogenic media. *Basrah Journal of Veterinary Research*.

[35] Merlino J., Siarakas S., Robertson G. J. (1996). Evaluation of CHROMagar orientation for differentiation and presumptive identification of gram-negativebacilli and *Enterococcus* species. *Journal of Clinical Microbiology*.

[36] Amaya E., Reyes D., Paniagua M. (2012). Antibiotic resistance patterns of *Escherichia coli* isolates from different aquatic environmental sources in Leon, Nicaragua. *Clinical Microbiology and Infection*.

[37] (2012). *Manual for the Phene-Plate System 2012*.

[38] CLSI (2010). *Performance Standards for Antimicrobial Susceptibility Testing: Nineteenth Informational Supplement, in M100-S1*.

[39] Thomas M., Fenske G. J., Antony L. (2017). Whole genome sequencing-based detection of antimicrobial resistance and virulence in non-typhoidal *Salmonella enterica* isolated from wildlife. *Gut Pathogens*.

[40] Zankari E., Hasman H., Cosentino S. (2012). Identification of acquired antimicrobial resistance genes. *Journal of Antimicrobial Chemotherapy*.

[41] Jia B., Raphenya A. R., Alcock B. (2017). CARD 2017: expansion and model-centric curation of the comprehensive antibiotic resistance database. *Nucleic Acids Research*.

[42] Hendricks R., Pool E. J. (2012). the effectiveness of sewage treatment processes to remove faecal pathogens and antibiotic residues. *Journal of Environmental Science and Health. Part A, Toxic/hazardous Substances & Environmental Engineering*.

[43] Morozzi G., Sportolari R., Caldini G. (1988). the effect of anaerobic and aerobic wastewater treatment on faecal coliforms and antibiotic-resistant faecal coliforms. *Zentralblatt für Bakteriologie, Mikrobiologie und Hygiene*.

[44] Ma L., Mao G., Liu J. (2013). Rapid quantification of bacteria and viruses in influent, settled water, activated sludge and effluent from a wastewater treatment plant using flow cytometry. *Water Science and Technology*.

[45] Ferguson A. S., Layton A. C., Mailloux B. J. (2012). Comparison of fecal indicators with pathogenic bacteria and rotavirus in groundwater. *Science of the Total Environment*.

[46] Gilbert R. (2000). Guidelines for the microbiological quality of some ready-to-eat foods sampled at the point of sale. PHLS advisory committee for food and dairy products. *Communicable Disease and Public Health*.

[47] Gruber J. S., Ercumen A., Colford J. M. (2014). Coliform bacteria as indicators of diarrheal risk in household drinking water: systematic review and meta-analysis. *PLoS One*.

[48] Levy K., Hubbard A. E., Nelson K. L. (2009). Drivers of water quality variability in northern coastal Ecuador. *Environmental Science & Technology*.

[49] Hrenovic J., Ivankovic T., Ivekovic D. (2017). the fate of carbapenem-resistant bacteria in a wastewater treatment plant. *Water Research*.

[50] Atashgahi S., Aydin R., Dimitrov M. R. (2015). Impact of a wastewater treatment plant on microbial community composition and function in a hyporheic zone of a eutrophic river. *Scientific Reports*.

[51] Gómez López M. J. (2010). *Waste Water Treatment Plants as Redistributors of Resistance Genes in Bacteria Water Pollution X*.

[52] Da Costa P. M., Vaz-Pires P., Bernardo F. (2006). Antimicrobial resistance in *Enterococcus* spp. isolated in inflow, effluent and sludge from municipal sewage water treatment plants. *Water Research*.

[53] Novo A., André S., Viana P. (2013). Antibiotic resistance, antimicrobial residues and bacterial community composition in urban wastewater. *Water Research*.

[54] Łuczkiewicz A., Jankowska K., Fudala-Książek S. (2010). Antimicrobial resistance of fecal indicators in municipal wastewater treatment plant. *Water Research*.

[55] Li W., Shi Y., Gao L. (2013). Occurrence, distribution and potential affecting factors of antibiotics in sewage sludge of wastewater treatment plants in China. *Science of the Total Environment*.

[56] Atterby C., Börjesson S., Ny S. (2017). ESBL-producing *Escherichia coli* in Swedish gulls-a case of environmental pollution from humans?. *PLoS One*.

[57] Nuangmek A., Rojanasthien S., Chotinun S. (2018). Antimicrobial resistance in ESBL-producing *Escherichia coli* isolated from layer and pig farms in Thailand. *Acta Scientiae Veterinariae*.

[58] Korzeniewska E., Harnisz M. (2013). Extended-spectrum beta-lactamase (ESBL)-positive *Enterobacteriaceae* in municipal sewage and their emission to the environment. *Journal of Environmental Management*.

[59] Yao Y., Lazaro-Perona F., Falgenhauer L. (2017). Insights into a novel blaKPC-2-encoding IncP-6 plasmid reveal carbapenem-resistance circulation in several *Enterobacteriaceae* species from wastewater and a hospital source in Spain. *Frontiers in Microbiology*.

[60] Blanch A. R., Belanche-Munoz L., Bonjoch X. (2006). Integrated analysis of established and novel microbial and chemical methods for microbial source tracking. *Applied and Environmental Microbiology*.

[61] Ahmed W., Neller R., Katouli M. (2005). Host species-specific metabolic fingerprint database for enterococci and *Escherichia coli* and its application to identify sources of fecal contamination in surface waters. *Applied and Environmental Microbiology*.

[62] Shaikh S., Fatima J., Shakil S. (2015). Antibiotic resistance and extended spectrum beta-lactamases: types, epidemiology and treatment. *Saudi Journal of Biological Sciences*.

[63] Zarfel G., Lipp M., Gürtl E. (2017). Troubled water under the bridge: screening of River Mur water reveals dominance of CTX-M harboring *Escherichia coli* and for the first time an environmental VIM-1 producer in Austria. *Science of the Total Environment*.

[64] Yamasaki S., Le T. D., Vien M. Q. (2017). Prevalence of extended-spectrum beta-lactamase-producing *Escherichia coli* and residual antimicrobials in the environment in Vietnam. *Animal Health Research Reviews*.

[65] Onnberg A., Mölling P., Zimmermann J. (2011). Molecular and phenotypic characterization of *Escherichia coli* and *Klebsiella pneumoniae* producing extended-spectrum beta-lactamases with focus on CTX-M in a low-endemic area in Sweden. *APMIS*.

[66] Bréchet C., Plantin J., Sauget M. (2014). Wastewater treatment plants release large amounts of extended-spectrum *β*-lactamase–producing *Escherichia coli* into the environment. *Clinical Infectious Diseases*.

[67] Canton R., Gonzalez-Alba J. M., Galan J. C. (2012). CTX-M enzymes: origin and diffusion. *Frontiers in Microbiology*.

[68] Kappell A. D., Denies M. S., Ahuja N. H. (2015). Detection of multi-drug resistant *Escherichia coli* in the urban waterways of Milwaukee, WI. *Frontiers in Microbiology*.

[69] Burmeister A. R. (2015). Horizontal gene transfer. *Evolution, Medicine, and Public Health*.

[70] von Wintersdorff C. J. H., Penders J., van Niekerk J. M. (2016). Dissemination of antimicrobial resistance in microbial ecosystems through horizontal gene transfer. *Frontiers in Microbiology*.

[71] Warnes S. L., Highmore C. J., Keevil C. W. (2012). Horizontal transfer of antibiotic resistance genes on abiotic touch surfaces: implications for public health. *mBio*.

[72] Brook I., Wexler H. M., Goldstein E. J. C. (2013). Antianaerobic antimicrobials: spectrum and susceptibility testing. *Clinical Microbiology Reviews*.

[73] Laffite A., Kilunga P. I., Kayembe J. M. (2016). Hospital effluents are one of several sources of metal, antibiotic resistance genes, and bacterial markers disseminated in sub-Saharan Urban rivers. *Frontiers in Microbiology*.

[74] Runcharoen C., Raven K. E., Reuter S. (2017). Whole genome sequencing of ESBL-producing *Escherichia coli* isolated from patients, farm waste and canals in Thailand. *Genome Medicine*.

[75] Fernando D. M., Tun H. M., Poole J. (2016). Detection of antibiotic resistance genes in source and drinking water samples from a first nations community in Canada. *Applied and Environmental Microbiology*.

[76] Lamba M., Ahammad S. Z. (2017). Sewage treatment effluents in Delhi: a key contributor of *β*-lactam resistant bacteria and genes to the environment. *Chemosphere*.

[77] Saleh A., Gottig S., Hamprecht A. (2018). Multiplex immunochromatographic detection of OXA-48, KPC and NDM carbapenemases: impact of the inoculum, antibiotics and agar. *Journal of Clinical Microbiology*.

[78] Pfaller M. A., Huband M. D., Mendes R. E. (2018). In vitro activity of meropenem-vaborbactam and characterization of carbapenem resistance mechanisms among carbapenem-resistant *Enterobacteriaceae* from the 2015 meropenem-vaborbactam surveillance program. *International Journal of Antimicrobial Agents*.

[79] O’Brien T. F., Stelling J. (2011). Integrated multilevel surveillance of the world’s infecting microbes and their resistance to antimicrobial agents. *Clinical Microbiology Reviews*.

