# Supplementary material for: Antimicrobial-Resistant Escherichia coli from Environmental Waters in Northern Colorado
Source: J Environ Public Health. 2019 Feb 18;2019:3862949. doi: 10.1155/2019/3862949 (PMC6397973; doi:10.1155/2019/3862949)
Supplement: Supplementary Materials — Figure S1: a map of Fort Collins, Colorado, and the surrounding area with sampling locations of environmental waters. Table S1: AMR profiles of the individual E. coli isolates examined with broth microdilution. Table S2: an expanded whole-genome sequence table that details all genes detected across wastewater E. coli isolates. [file 3862949.f1.zip › 3862949.f1/Final JEPH Supplementary Table S1_JEPH_2637083.docx]

**Table S1** Antimicrobial resistance profiles of *E. coli* examined with broth microdilution.

| **Location** | Amikacin | Gentamicin | Tobramycin | Amoxicillin/Clavulanic Acid | Ampicillin | Cefalexin | Cefovecin | Cefpodoxime | Ceftiofur | Imipenem | Piperacillin | Chloramphenicol | Marbofloxacin | Enrofloxacin | Nitrofurantoin | Tetracycline | Trimethoprim/ Sulfamethoxazole |
| --- | --- | --- | --- | --- | --- | --- | --- | --- | --- | --- | --- | --- | --- | --- | --- | --- | --- |
| **Sewage** | S | S | S | S | S | S | S | S | S | S | S | S | S | S | S | S | S |
|  | S | S | S | R | R | R | R | R | I | R | R | S | R | R | S | R | S |
|  | S | S | S | S | S | S | S | S | S | S | S | S | S | S | S | S | S |
|  | S | R | I | S | R | S | S | S | S | S | R | R | S | I | S | R | S |
|  | S | S | S | S | S | S | S | S | S | S | S | I | S | S | S | S | S |
|  | S | R | R | R | R | R | R | R | R | R | R | S | S | I | S | R | S |
|  | S | S | S | S | S | S | S | S | S | S | S | S | S | S | S | S | S |
|  | S | S | S | S | S | S | S | S | S | S | S | S | S | S | S | S | S |
|  | S | S | S | S | S | S | S | S | S | S | S | I | S | S | S | S | S |
|  | S | S | S | S | S | S | S | S | S | S | S | S | S | S | S | S | S |
|  | S | S | S | S | S | S | S | S | S | S | S | S | S | S | S | S | S |
|  | S | S | S | R | R | R | R | R | R | R | R | S | S | S | S | S | S |
|  | S | S | S | S | S | S | S | S | S | S | S | S | S | S | S | S | S |
|  | S | S | S | R | R | R | R | R | R | R | R | S | S | I | S | S | S |
|  | S | S | S | S | R | R | R | R | I | S | R | S | R | R | S | R | S |
|  | S | S | S | R | R | R | R | R | R | R | R | S | S | S | S | S | S |
|  | S | S | S | S | S | S | S | S | S | S | S | S | S | S | S | S | S |
| **WWTP Influent** | S | S | S | S | S | S | S | S | S | S | S | S | S | S | S | R | S |
|  | S | S | S | R | R | R | R | R | R | R | R | S | S | I | S | S | R |
|  | S | S | S | R | R | R | R | R | R | R | R | S | S | I | S | S | S |
|  | S | S | S | S | S | S | S | S | S | S | S | S | S | S | S | S | S |
|  | S | S | S | S | S | S | S | S | S | S | S | S | S | S | S | S | S |
|  | S | S | S | S | S | S | S | S | S | S | S | S | S | S | S | S | S |
|  | S | S | S | R | R | R | R | R | R | R | R | S | S | S | I | S | R |
|  | S | S | S | R | R | R | R | R | R | R | R | I | S | S | S | S | S |
|  | S | S | S | I | R | S | S | S | S | S | R | R | R | R | S | S | S |
|  | S | S | S | S | S | S | S | S | S | S | S | S | S | S | S | S | S |
| **WWTP-Effluent** | S | S | S | S | S | S | S | S | S | S | S | S | S | S | S | S | S |
|  | S | R | R | R | R | R | R | R | R | R | R | S | S | I | S | R | S |
| **Surface Water** | S | S | S | S | S | S | S | S | S | S | S | S | S | S | S | S | S |
|  | S | S | S | S | S | S | S | S | S | S | S | S | S | S | S | S | S |
|  | S | S | S | S | S | S | S | S | S | S | S | S | S | S | S | S | S |
|  | S | S | S | S | S | S | S | S | S | S | S | S | S | S | S | S | S |
|  | S | S | S | S | S | S | S | S | S | S | S | S | S | S | S | S | S |

R = Resistant; I= Intermediate; S= Susceptible
