# Supplementary figures and images for: Antimicrobial-Resistant Escherichia coli from Environmental Waters in Northern Colorado
Source: J Environ Public Health. 2019 Feb 18;2019:3862949. doi: 10.1155/2019/3862949 (PMC6397973; doi:10.1155/2019/3862949)

**
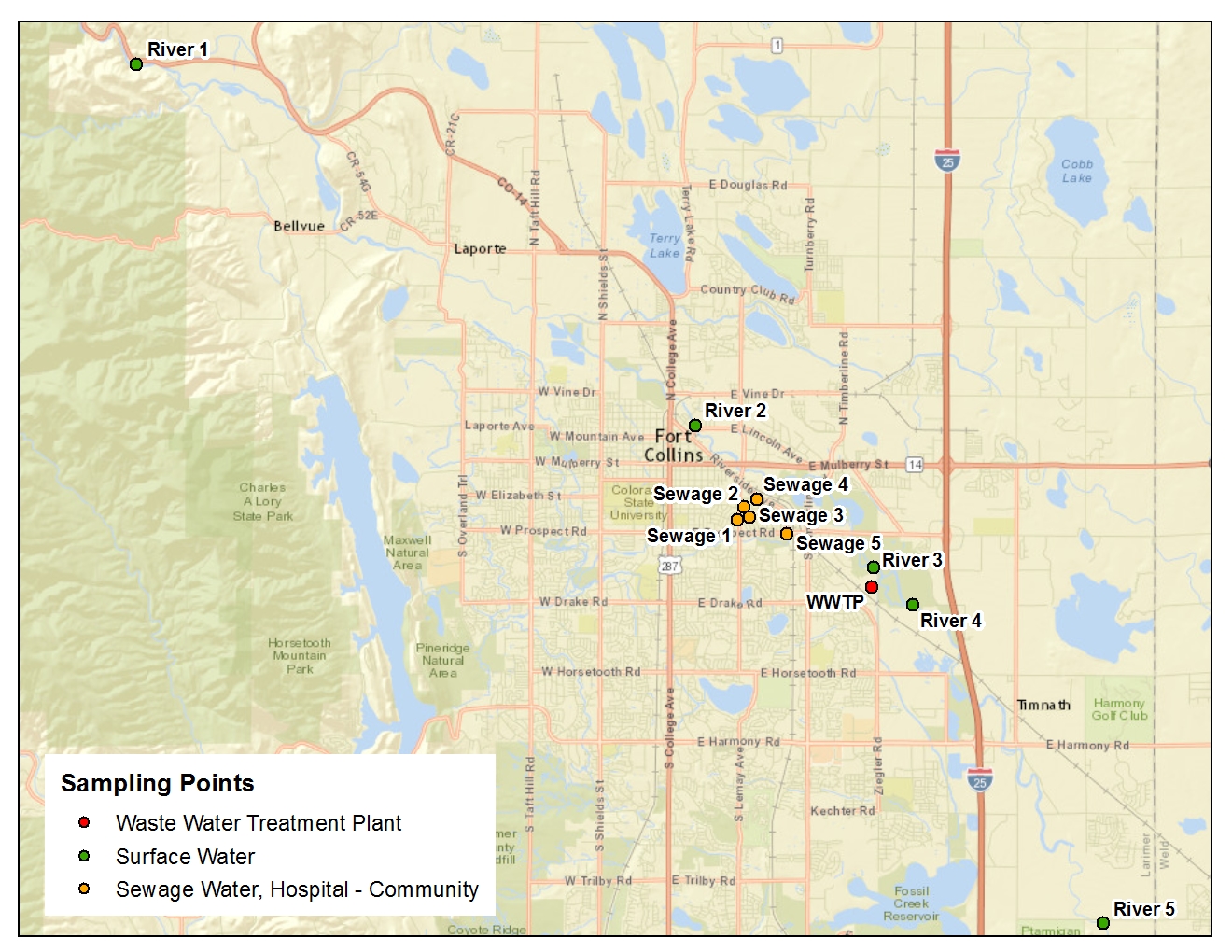
FIG S1** Environmental water sampling locations in and around Fort Collins, Colorado.

Supplement: Supplementary Materials — Figure S1: a map of Fort Collins, Colorado, and the surrounding area with sampling locations of environmental waters. Table S1: AMR profiles of the individual E. coli isolates examined with broth microdilution. Table S2: an expanded whole-genome sequence table that details all genes detected across wastewater E. coli isolates. [file 3862949.f1.zip › 3862949.f1/Final JEPH Supplementary Figure S1_JEPH_2637082.docx]
